# Supplementary material for: Development of metastasis and survival prediction model of luminal and non-luminal breast cancer with weakly supervised learning based on pathomics
Source: PeerJ. 2025 Jan 21;13:e18780. doi: 10.7717/peerj.18780 (PMC11759606; doi:10.7717/peerj.18780)
Supplement: Supplemental Information 4 [file peerj-13-18780-s004.docx]

## Supplementary

**Supervise Learning Results**

#### **Patch Level Efficiency**

In our study, models based on supervised algorithms demonstrated an improvement in AUC on the test set compared to those using weakly supervised algorithms. However, due to differences in training samples (290319[train]+133000[test] in supervised learning and 530240 [train]+269582[test] in unsupervised learning), the impact of this improved recognition on downstream tasks requires further validation.

| **Model Name** | **Acc** | **AUC** | **95% CI** | **Sensitivity** | **Specificity** | **PPV** | **NPV** | **Cohort** |
| --- | --- | --- | --- | --- | --- | --- | --- | --- |
| densenet121 | 0.930 | 0.983 | 0.9824-0.9831 | 0.948 | 0.900 | 0.939 | 0.915 | train |
| densenet121 | 0.774 | 0.818 | 0.8150-0.8200 | 0.832 | 0.643 | 0.840 | 0.629 | test |
| inception_v3 | 0.915 | 0.975 | 0.9750-0.9759 | 0.937 | 0.880 | 0.926 | 0.897 | train |
| inception_v3 | 0.788 | 0.824 | 0.8214-0.8263 | 0.941 | 0.444 | 0.792 | 0.769 | test |
| resnet50 | 0.942 | 0.988 | 0.9877-0.9882 | 0.957 | 0.918 | 0.949 | 0.930 | train |
| resnet50 | 0.777 | 0.823 | 0.8201-0.8250 | 0.835 | 0.647 | 0.842 | 0.635 | test |

Supplementary Table 1: Displays the patch-level accuracy and AUC scores of each model.

Our results indicate that, for ER identification, the Inception V3 model exhibits particularly robust patch recognition capabilities. Consequently, both our PLH and BoW pipelines incorporate multiple instance learning feature aggregation based on the recognition outcomes of Inception V3.


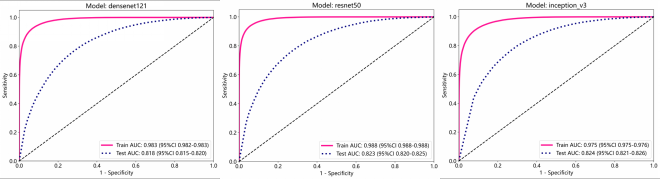


Supplementary Figure 1: The ROC curves for each model's performance on the three datasets, namely Densenet121, ResNet50, and Inception_v3, arranged from left to right.

### Metastasis Analysis

| **Model name** | **Accuracy** | **AUC** | | **95% CI** | **Sensitivity** | **Specificity** | **PPV** | **NPV** | **Task** |
| --- | --- | --- | --- | --- | --- | --- | --- | --- | --- |
| RandomForest | 0.829 | | 0.888 | 0.7997 - 0.9768 | 0.850 | 0.824 | 0.531 | 0.959 | Train |
| RandomForest | 0.604 | | 0.707 | 0.4157 - 0.9991 | 0.667 | 0.600 | 0.100 | 0.964 | Test |
| XGBoost | 0.876 | | 0.828 | 0.7270 - 0.9295 | 0.600 | 0.941 | 0.706 | 0.909 | Train |
| XGBoost | 0.938 | | 0.744 | 0.4929 - 0.9960 | 0.000 | 1.000 | 0.000 | 0.937 | Test |
| LightGBM | 0.714 | | 0.846 | 0.7609 - 0.9308 | 0.750 | 0.706 | 0.375 | 0.923 | Train |
| LightGBM | 0.562 | | 0.733 | 0.5181 - 0.9486 | 0.667 | 0.556 | 0.091 | 0.962 | Test |

Supplementary Table2: Metrics in train and test cohort in predicting risk of metastasis.


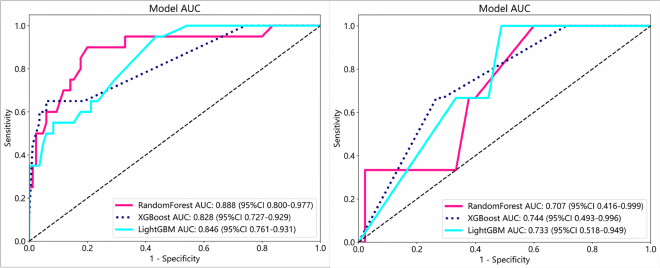


Supplementary Fig 2: The ROC in train cohort (Left) and test cohort (right) in predicting risk of metastasis.

### Survival Analysis

**C-index**: The nomogram employing the Cox algorithm was utilized to integrate clinical features and the radiomic signature, demonstrating superior performance. We evaluated the model results using the C-index, which yielded values of 0.912 and 0.652 in the training and testing sets, respectively.


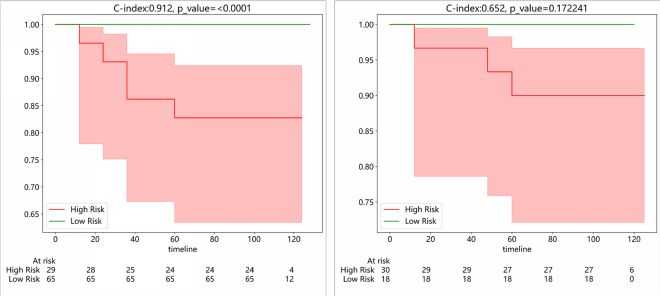


Supplementary Fig3. Kaplan-Meier (KM) analysis for the training cohort and the test cohort
